# How Does Epstein–Barr Virus Interact With Other Microbiomes in EBV-Driven Cancers?

**DOI:** 10.3389/fcimb.2022.852066

**Published:** 2022-02-23

**Authors:** Yuxi Wen, Huan Xu, Juan Han, Runming Jin, Hongbo Chen

**Affiliations:** Department of Pediatrics, Union Hospital, Tongji Medical College, Huazhong University of Science and Technology, Wuhan, China

**Keywords:** Epstein–Barr virus, reactivation, EBV-driven cancer, microbiome, host–microbiota interaction

## Abstract

The commensal microbiome refers to a large spectrum of microorganisms which mainly consists of viruses and bacteria, as well as some other components such as protozoa and fungi. Epstein–Barr virus (EBV) is considered as a common component of the human commensal microbiome due to its spread worldwide in about 95% of the adult population. As the first oncogenic virus recognized in human, numerous studies have reported the involvement of other components of the commensal microbiome in the increasing incidence of EBV-driven cancers. Additionally, recent advances have also defined the involvement of host–microbiota interactions in the regulation of the host immune system in EBV-driven cancers as well as other circumstances. The regulation of the host immune system by the commensal microbiome coinfects with EBV could be the implications for how we understand the persistence and reactivation of EBV, as well as the progression of EBV-associated cancers, since majority of the EBV persist as asymptomatic carrier. In this review, we attempt to summarize the possible mechanisms for EBV latency, reactivation, and EBV-driven tumorigenesis, as well as casting light on the role of other components of the microbiome in EBV infection and reactivation. Besides, whether novel microbiome targeting strategies could be applied for curing of EBV-driven cancer is discussed as well.

## Introduction

Epstein–Barr virus (EBV) could be considered as a component of the human microbiome as a consequence of its roughly sustaining 95% of the adult populations worldwide, and majority of it persists lifelong as an asymptomatic carrier (Dreyfus, 2013; Young et al., 2016; Connolly et al., 2021). In addition, EBV is the first identified human oncogenic virus (De Martel et al., 2020) which was discovered 50 years ago (Young et al., 2016). The importance of EBV reactivation is emphasized in the progression of EBV-driven carcinogenesis since the antibodies for capsid antigen and EBV-DNase of EBV were observed to be increased prior to the tumorigenesis in nasopharyngeal carcinoma (NPC) (Chien et al., 2001). Therefore, factors which might induce the EBV reactivation will be emphasized in this review for the pathogenesis of EBV-driven cancers.

The commensal microbiome refers to the diverse microorganisms, which consist mainly of bacteria and virus, as well as other components such as archaea, fungi, and protozoa that colonize barrier surfaces of different niches of mammals, such as the skin, vaginal, upper respiratory, and gastrointestinal tracts (Lloyd-Price et al., 2016; Barko et al., 2018). For the past few years, microbiome studies focus mainly on the composition and function of bacteria and archaea (Carding et al., 2017; Nikolich-Zugich et al., 2017). However, recent virome studies emphasized that viruses, which are abundant in divergent tissues (such as oral cavity, skin, gut, and blood) as well as in the feces of individuals in sickness and in health (Norman et al., 2015; Neil and Cadwell, 2018; Schmidt, 2018; Clooney et al., 2019), are the largest proportion of the human microbiome instead of bacteria (Wylie et al., 2012). The virome of human, which consists of diverse viruses that could infect not only eukaryotic cells but also prokaryotic cells (Handley, 2016), is an important factor in host health and diseases (Cadwell, 2015).

As EBV is sustained in almost all adult humans, it is puzzling why only a few of them evolved to induce malignant transformation. Some cooperative triggers must occur in the tumorigenesis of EBV-driven cancers. In the 1960s, the role of the commensal microbiome in modulating virus infections was first suggested (Robinson and Pfeiffer, 2014). The susceptibility of host to virus infection was enhanced in germ-free (GF) mice. Over the past few years, host–microbiota interactions have been reported to be fundamental for the regulation and maintenance of the mammalian immune system (Belkaid and Hand, 2014; Belkaid and Harrison, 2017; Rowan-Nash et al., 2019), which could be the implication for how we understand the persistence and reactivation of EBV, as well as the progression of EBV-associated cancers. In the present review, we aimed to discuss the impact of the microbiome on EBV infection. In addition, we focus on how the microbiome reactivates latently infected EBV and pertain to the etiology of EBV-driven cancers in patients who could be with asymptomatic life-long infection.

## EBV Infection and EBV-Associated Cancers

**Figure 1:**
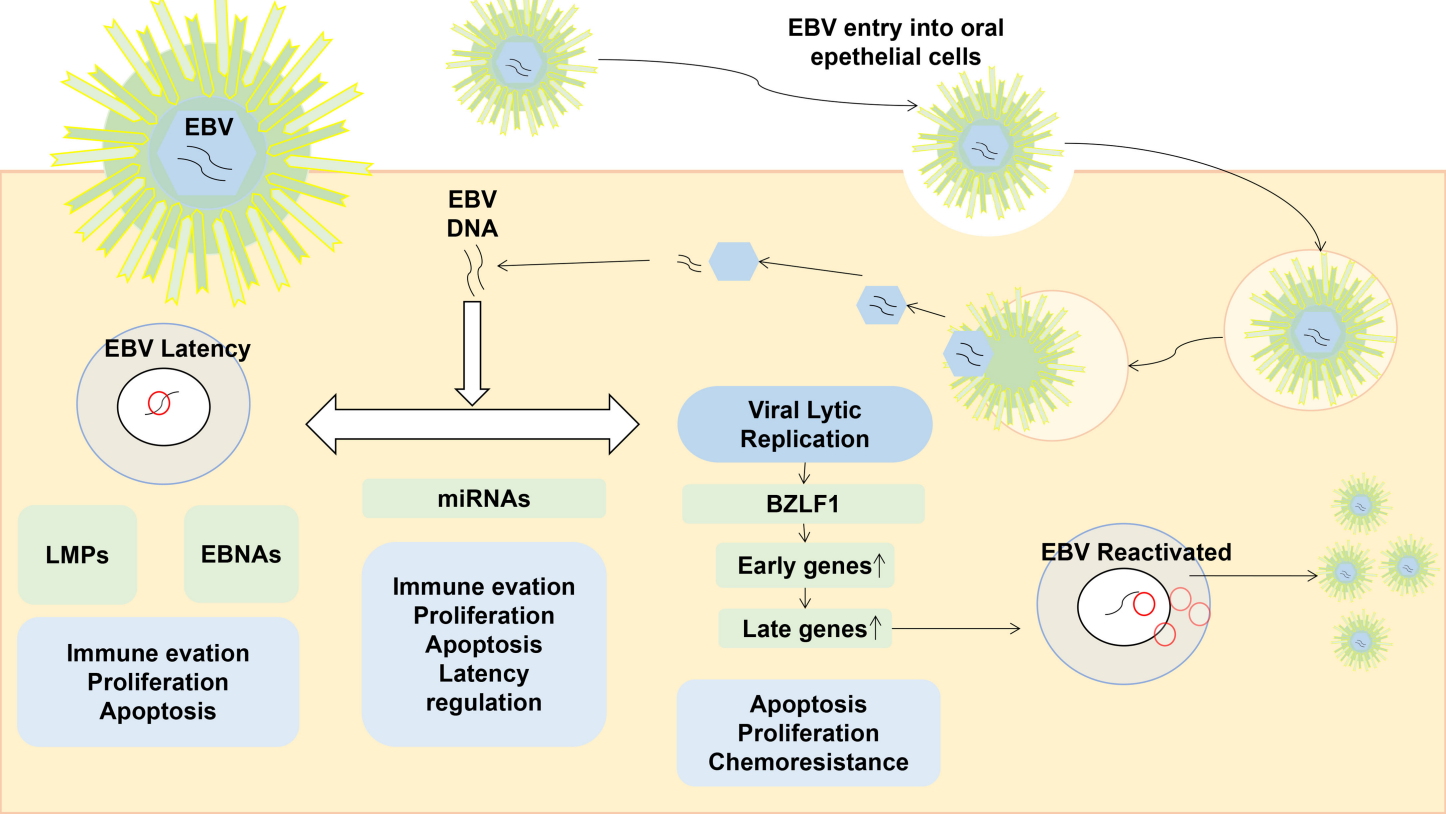
The life cycle of EBV and the role of gene expression in a latent or lytic phase. EBV is normally spread by saliva, then it first enters into the oropharyngeal epithelia cells. During the latent infection phase, EBV DNA persists by attaching to the host chromosomes, and the expression of BZLF1 is vital in the EBV reactivation which would promote the expression of early and late lytic genes. Reactivated EBV are cleaved, packaged, and released outside of the infected cell.

EBV is a globally spread virus that infects and persists lifelong in about 95% of the world’s population (Torniainen-Holm et al., 2018). The life cycle of EBV includes a latent infection phase, during which the virus persists by attaching to the host chromosomes, and a lytic replication phase, predominantly occurring in oropharyngeal epithelial cells (Hochberg et al., 2004) (shown as **Figure 1**). During the latency, a few EBV viral promotors are dynamically regulated, and the latency of EBV is classified from latency 0 to 3 according to the differential expressed sets of viral genes in a cell-dependent manner (Woisetschlaeger et al., 1990; Ohga et al., 2002). The latent EBV genomes are spontaneously reactivated by various stimuli, including pathogenic infections and other commensal microbiomes. The virion episome is spliced to linear and released from infected cells during the transformation of EBV latency into a lytic/replicative phase.

EBV harbors high tumorigenic potential preferentially infecting B-cells, T-cells, natural killer (NK) lymphocytes, and epithelial cells, steadily promoting the uncontrolled proliferation of infected cells (Miller and Lipman, 1973a; Miller and Lipman, 1973b), leading to a wide spectrum of EBV-positive cancers (Cai et al., 2015). According to the EBV-infected cell type involved in the tumorigenesis, the divergent EBV-driven cancer could be grouped into three mainly groups, including lymphoproliferative disorders (LPD) (Heslop, 2020), epithelial malignancies (Yamaguchi et al., 2018), and lymphoepithelioma-like carcinoma (LELC) (Ose et al., 2021) (**Table 1**).

**Table 1:** The EBV-driven cancers and the involvement of coinfections.

Classification	Infected cell type	Tumor	Coinfection	Latency	References	Incidence of EBV infection	Detection method
**Lymphoproliferative disorders**	**B cell**	**Diffuse large B cell lymphoma (DLBCL)**	**HIV**	**Latency 1/2/3-**	(Ozsan et al., 2013)	**30–50%**	***In situ* hybridization**
		**Primary effusion lymphoma**	**KSHV** **HIV**	**Latency 1**	(Bigi et al., 2018)	**70–100%**	
	**T cell**	**Burkitt lymphoma**	**Malaria** **HIV**	**Latency 2/3**	(Moormann and Bailey, 2016; Heslop, 2020)	**>90%**	**-**
		**Hodgkin’s lymphoma**	**HIV**	**Latency 2**	(Pánisová et al., 2022)	**27.7%**	**Serologies**
		**Non-Hodgkin’s lymphoma**	**HIV**		(Piriou et al., 2005)	**-**	**-**
	**NK cell**	**Aggressive NK-cell leukemia (ANKL)**			(Ishida, 2018)	**~90%**	
		**Extranodal NK/T-cell lymphoma, nasal-type (ENKTL)**		**Latency 1/2**	(Mao et al., 2012)		***In situ* hybridization**
**Epithelial malignancies**	**Nasopharyngeal epithelium**	**Undifferentiated NPC**	**HPV**	**Latency 2**	(Tsao et al., 2017)		
	**Gastric epithelial cells**	**Gastric adenocarcinomas**	**-**	**Latency 1/2**	(Okabe et al., 2020)	**8–10%**	
**Lymphoepithelioma-like carcinoma (LELC)**		**Gastric carcinoma**		**Latency 1/2**	(Song et al., 2021)	**90%**	
		**Oropharyngeal carcinomas**	**HPV**		(Blanco et al., 2021)		
	**Breast epithelial cells**	**Breast cancer**	**HPV**	**Latency 2**	(Sinclair et al., 2021; Gupta et al., 2021)	**0–31%**	
		**Thyroid**			(Shimakage et al., 2003)		**mRNA *in situ* hybridization, indirect immunofluorescence staining, polymerase chain reaction (PCR)**
	**Salivary gland epithelial cells**	**Salivary gland carcinomas - LELCs**	**-**	**-**	(Tsai et al., 1996)	**12.5%**	***In situ* hybridization**
	**Renal epithelial cells**	**Renal cell carcinoma**			(Shimakage et al., 2007; Kryst et al., 2020)	**29.6%**	**mRNA *in situ* hybridization and indirect immunofluorescence staining**
	**Prostate epithelial cells**	**Prostate cancer**	**HPV**		(Whitaker et al., 2013; Nahand et al., 2021)	**24-30%**	***In situ* polymerase chain reaction (IS-PCR) and standard liquid PCR;** **enzyme-linked immunosorbent assay (ELISA) and quantitative real-time polymerase chain reaction**
	**Urothelial epithelial cells**	**Upper urinary tract urothelial carcinomas**		**-**	(Dere et al., 2020)	**29.5%**	**Chromogenic *in situ* hybridization** **Real-time PCR**

## Maintenance of EBV Latency

The latency of EBV has been grouped according to the immune status of the patient and expression of EBV proteins (Carbone et al., 2008; Taylor et al., 2015), and the differences among these latency groups determine the treatment responses of EBV. Latency 0 only expresses the EBV-encoded messenger RNA (EBER) and BamH1-a rightward reading frame transcript (BART) in cells of healthy previously infected individuals. In Burkitt lymphoma, gastric cancer, HIV-associated diffuse large B-cell lymphoma (HIV-DLBCL), and approximately two-thirds of nasopharyngeal carcinoma, EBV exists in a latency 1 pattern and only expresses EBV-nuclear antigen 1 (EBNA1), EBER, and BARTs (Tempera and Lieberman, 2014), thereby evading immune responses to EBV (Burkitt, 1958; Arvey et al., 2015). Latency 3, which persists only in severely immunocompromised hosts such as EBV-positive post-transplant lymphoproliferative disorder (PTLD), expresses all virus-specific latent nuclear antigens and membrane proteins (Gottschalk et al., 2005; Lacasce, 2006). For latency 2, late membrane protein-1 (LMP1) is expressed in addition on the basis of latency 1, and this phase has been observed in Hodgkin, some nasopharyngeal carcinoma, and so on (Sekihara et al., 2014). As a consequence of the restricted antigen expression, EBV-CTLs (Bollard et al., 2014) which act well in latency 2 fail in latency 1 tumors.

During the viral infections, many viruses would enter the latency state with their genetic materials integrated into the genome of the infected cells (Nikolich-Zugich et al., 2017) and regulate the host immune system (Virgin, 2014). Viral infections are reported to modulate the host immune system by stimulating the production and release of a variety of cytokines, including interferon and other cytokines such as interleukin (IL)-10 (Nikolich-Zugich et al., 2017). As for the role of EBV in infected hosts, EBV and related herpesvirus are assumed to encode a copy of human cytokines that regulate the host immune system to satisfy their own colonization and expansion needs (Irons and Le, 2008; Slobedman et al., 2009). A human IL-10-like protein that acts in the TH2 family was reported to be encoded by EBV (Hsu et al., 1990; Macneil et al., 1990; Moore et al., 1990). The LMP1 expression of EBV could act as the tumor necrosis factor (TNF) receptors to transmit the growth signals through TNF-receptor-associated factors (TRAFs) (Liebowitz, 1998) and thus is vital for the EBV-induced tumorigenesis (Iwakiri et al., 2013). EBER was reported to act as a substitute of interferon and induce the expression of IL-9/10 and insulin-like growth factor, resulting in the promotion of EBV-infected cells growth (Kitagawa et al., 2000; Iwakiri et al., 2003; Yang et al., 2004; Iwakiri et al., 2005).

It is vital to elucidate the mechanisms involved in the EBV reactivation since the chronic EBV reactivation is considered as a key mechanism in the pathogenesis of EBV-driven or -associated cancers.

## Reactivation of Latent EBV

Despite that mechanisms for EBV latency have been clarified, those required to reactivate latent EBV have not yet been elucidated well. Since reactivation of latent EBV is associated with EBV-driven cancers (Chien et al., 2001), the further understanding of the mechanisms that promote EBV reactivation is of great significance for revealing the pathobiology of EBV-driven cancers and developing novel therapies against it. The epigenetic regulation such as deacetylation by histone deacetylases (HDACs) is reported to play central roles in viral latency and reactivation (Li et al., 2007; Berger, 2007). EBV tends to establish a latency state in infected cells, and once transformed to the lytic replication cycle, cells are regulated by the “open” and “closed” conformations of chromatin (Tsurumi et al., 2005; Murata et al., 2021). For the maintenance and disruption of EBV latency, HAT and HDAC take part in the post-translational modification (hypoacetylation) of DNA-associated histone in the BZLF1 promoter (Liu et al., 1997a; Liu et al., 1997b; Gruffat et al., 2002). Besides, methylation also plays an important role in EBV latency maintenance (Murata et al., 2012; Imai et al., 2014).

During lytic infection, the viral genome of EBV is amplified up to 1,000-fold and expresses a variety of EBV genes to maintain the cell cycle progression in the S-phase which is necessary for viral replication (Tsurumi et al., 2005). The expression of the BamHI Z/R fragment leftward open reading frame 1 (BZLF1/BRLF1) genes can induce the lytic cycle by cascade transactivating both early and late EBV genes (Speck et al., 1997; Tsurumi et al., 2005; Murata et al., 2021). For the final step of the EBV lytic cycle, the virion genome is replicated, cleaved, packaged, and released to infect other susceptible cells (Tsurumi et al., 2005). During the EBV infection, the expressions of LMP1 and EBNA2 (Sekihara et al., 2014) during the lytic/replicative phase are considered as oncogenic; thus, regulation of LMP1 and EBNA could suppress the tumorigenesis.

## Implication of Diverse Microbial Interacts With EBV in EBV-Driven Cancers

### Coinfections of Other Components of Microbiome and EBV in EBV-Driven Cancers

Despite that EBV can transform lymphocyte and other cells to tumorigenesis, it is puzzling why the prognosis of patients with EBV-positive gastric adenocarcinoma or HL is often better than that of patients with EBV-negative cancers (Van Beek et al., 2004; Keegan et al., 2005), which still has not been elucidated. Previously, the interplays between EBV and other components of the microbiome which could contribute to EBV-driven cancers will be discussed here.

EBV-associated cancer is often associated with coinfections that regulate the host immune system. The higher EBV loads were reported in the peripheral blood of HIV-infected individuals (Yan et al., 2018), which leads to the development of EBV-associated cancers, such as lymphomas (Dolcetti et al., 1995; Vaghefi et al., 2006). Investigations also revealed an elevated EBV load in continuously *Plasmodium falciparum*-exposed children (Moormann et al., 2005; Njie et al., 2009). Using the 16S gene ribosomal RNA sequencing, a divergent expression of the gut microbiota KEGG functional pathway was described in the fecal samples of patients with EBVaGC, when compared with those with EBVnGC (Wang et al., 2019a). The coinfection with the dengue virus resulted in an increasing EBV replication in the blood cells (Deng et al., 2021). EBV has also been reported to coinfect with other secondary human pathogens such as HCMV (Jakovljevic et al., 2015), influenza virus, adenovirus, and severe acute respiratory syndrome coronavirus 2 (SARS-CoV-2), (Wang et al., 2010; Nadeem et al., 2021).

### Coinfections of Other Components of Microbiome Could Induce the EBV Reactivation and Lead to EBV-Driven Cancers

The mechanistic studies for the promotion of EBV reactivation and EBV-driven tumorigenesis by coinfections have been explored. When the hospitalized patients were coinfected with EBV and HCV, the immune responses could be dampened (Shoman et al., 2014). The PI3K signaling pathway was first reported to play a role in DENV-2’s reactivation of EBV (Deng et al., 2021). Studies suggested that cytotoxic lymphocytes can prevent the tumorigenesis of asymptomatic EBV carriers since a higher viral load and the latency III infection program were induced in the circumstances that the CD8+/CD4+ T cells and NK cells were inhibited or depleted (Murer et al., 2019; Münz, 2021). The regulation of host CD8+/CD4+ T cells and NK cells by coinfected pathogens has also been explored in EBV-driven cancers.

#### HPV Coinfects With EBV Leading to Carcinomas

HPV is reported to cause a large spectrum of carcinomas, and in some cases, it coinfects with EBV (Polz-Gruszka et al., 2015; Kienka et al., 2019). It still remains to be explored whether the coinfection is the etiology or just a phenomenon which is not causality. HPV/EBV coinfection was reported to present in some of prostate cancer (PCa) cases (Nahand et al., 2021), oral carcinogenesis (Blanco et al., 2021), cervical cancer (Feng et al., 2021), breast cancer (Nagi et al., 2021), and nasopharyngeal carcinoma (NPC) (Blanco et al., 2021) (shown in **Table 1**). The cytokine expression profile of PCa cases with HPV and EBV coinfection was quite different from that only infected with HPV or EBV. The differential expression profile suggests that HPV and EBV coinfection could be an etiology for the development of PCa. EBV could also play an important role in the integration of the HPV genome (Nahand et al., 2021). When co-expressed with low-risk HPV E6/E7 (HPV 6/11), EBV LMP-1 failed to induce the malignant transformation as that co-expressed with high-risk HPV E6/E7 (in primary mouse embryonic fibroblast (MEF) cells (Shimabuku et al., 2014; Blanco et al., 2021). However, the co-expression was proved to lead to precancerous lesions (Uehara et al., 2021) since it promoted the accumulation of DNA damage-related somatic mutations.

#### Malaria Coinfects With EBV Leading to Endemic Burkitt Lymphoma

For the past few years, coinfection of *Plasmodium falciparum* and EBV has been described to lead to the commonest pediatric cancer—endemic Burkitt lymphoma (eBL)—in equatorial Africa (Moormann and Bailey, 2016; Quintana et al., 2020) (shown as **Table 1**). Once infected with *Plasmodium falciparum*, an increased viral load could be induced by reactivating EBV and impairing the IFN-γ signal in children (Moormann et al., 2005; Moormann et al., 2007). Additionally, the repeated or prolonged malaria infection was also considered to disturb the immune surveillance directed by EBV-specific T/NK cell (toward EBNA1) in eBL (Forconi et al., 2018; Forconi et al., 2021). Malaria infection was known to activate EBV in affected children (Reynaldi et al., 2016) and induce the EBV replication by initiating the B-cell receptor (BCR) signal pathway (Chêne et al., 2007). Coinfection with *Plasmodium falciparum* was reported to induce infected B cells into a differential phase, resulting in the occurrence of c-myc translocation.

#### KSHV Coinfects With EBV in Primary Effusion Lymphoma and Kaposi Sarcoma

Kaposi sarcoma-associated herpesvirus (KSHV) which belongs to the γ-herpesvirus also coinfects with EBV in Kaposi sarcoma and primary effusion lymphoma (PEL) (2019; Mariggiò et al., 2017) (shown as **Table 1**). The simultaneous coinfection of EBV and KSHV was believed to promote the sustenance of KSHV both *in vivo* (Mchugh et al., 2017; Caduff et al., 2021 *)* and *in vitro* (Bigi et al., 2018). Additionally, the gene expression profile of PEL with coinfection of KSHV and EBV was divergent from that found in lymphomas which only carry EBV (Mchugh et al., 2017; Caduff et al., 2021). When coinfected with EBV in B cells, KSHV seemed to reactivate EBV (Mchugh et al., 2017). The fact that KSHV-coinfected EBV which was lytic replication deficient failed to induce lymphoma in mice favors that EBV reactivation is vital for EBV-driven tumorigenesis (Mchugh et al., 2017; Caduff et al., 2021). Besides for reactivating EBV in the host, KSHV coinfection was also reported to influence the NK cell differentiation (destined to CD56-negative NK cells) so as to assist replication as well as expansion pf lytic EBV (Caduff et al., 2021; Alari-Pahissa et al., 2021; Pánisová et al., 2022).

#### HIV Coinfects With EBV Contributing to B Cell Lymphoma

The coinfection of human immunodeficiency virus (HIV) and EBV exists in a large variety of lymphoma such as Burkitt lymphoma, Hodgkin’s lymphoma, diffuse large B cell lymphoma, PEL, and primary CNS lymphoma (Verdu-Bou et al., 2021) (shown as **Table 1**). The depletion of CD4+ T cells and senescence of CD8+ T cells are common in HIV carriers, which could contribute to the deficiency of EBV-specific T cell immune responses in EBV-driven lymphoma (Piriou et al., 2005; Hernández et al., 2018). The EBV control was reported to be compromised in EBV-driven lymphoma which coinfected with HIV due to the deficient EBNA1-specific CD4+/CD8+ T cells (Münz et al., 2000; Piriou et al., 2005; Mavilio et al., 2005), as well as the differentiation of NK cells to a CD56-negative NK cell without protective effects (Mavilio et al., 2005; Cao et al., 2021) in HIV carriers. Besides, the induction of the apolipoprotein B mRNA-editing enzyme, catalytic polypeptide-like (APOBEC) family, which was clarified as a DNA-modifying enzyme in HIV coinfection, might be another trigger for EBV-driven lymphoma by somatic mutation accumulation (Venkatesan et al., 2018). Furthermore, the cooperation between HIV and EBV in HIV-related lymphoma has been speculated, with HIV likely contributing to the generation of a permissive microenvironment for EBV infection, and the differentiation and survival of infected B-cells (Verdu-Bou et al., 2021).

## Other Commensal Microbiome Could Interact With EBV in EBV-Driven Cancer by Regulating the Immune System

The depletion of CD8+/CD4+ T cells and NK cells is reported to promote the occurrences of EBV-driven cancers (Murer et al., 2019; Münz, 2021), and extra evidence has also been claimed that the coinfection of EBV and some pathogen could have induced the EBV reactivation and impaired the EBV-targeted immune cell response. The commensal microbiota is considered as fundamental for the development of secondary lymphoid structures, as well as the differentiation, maturation, and function of T and B cells including virus-specific effector CD4^+^ and CD8^+^ T cells, FoxP3^+^ CD4^+^ T regulatory cells (Tregs) and Th17 cells, CD4^+^ helper T cells, and B cells (Hill and Artis, 2010; Furusawa et al., 2013; Khosravi et al., 2014; Josefsdottir et al., 2017; Luu et al., 2018; Zhao and Elson, 2018; Lee et al., 2021). It is reasonable to believe that other commensal microbiota coinfection with EBV could also be a trigger for EBV-driven cancer.

The disturbance of commensal microbiota is reported to result in impaired lymphoid tissue development and alter susceptibility to infectious diseases (Abt and Artis, 2009; Atarashi et al., 2011; Pickard et al., 2017). For example, the colonization of segmented filamentous bacteria (SFB) in the intestine is associated with increased CD4^+^ T helper 17 cells in the intestine (Wang et al., 2019b). The oral colonization of *Lactobacillus paracasei* has also been reported to induce an increased number of tissue resident and circulatory myeloid cells in mice lungs (Belkacem et al., 2017).

### The Commensal Microbiota Is Likely to Prime Type I IFN-Dependent Antiviral Immune Responses in EBV Infection

The binding of pattern recognition receptors (PRRs) to conserved ligands of commensal microbiota which are termed microbe-associated molecular patterns (MAMPs) is reported to shape and modulate host immune responses (Chu and Mazmanian, 2013). The MAMPs of virus, bacteria, protozoa, and fungi could bind to specific PRR, thus priming the type I IFN response (Ito et al., 1976; Abt et al., 2012; Ganal et al., 2012), which is the central component of virus control (Forero et al., 2019), and the production of divergent cytokines such as TNF-α and IL-6 (Ma et al., 2018; Winkler and Thackray, 2019). For example, in mice treated with antibiotics, the type I IFN response was diminished in peritoneal macrophages (MO) (Abt et al., 2012), resulting in the impaired ability to stimuli such as lipopolysaccharide (LPS), influenza virus (IAV), and lymphocytic choriomeningitis virus (LCMV).

### The Possible Role of the Commensal Microbiota in Priming Cell-Mediated Innate and Adaptive Immune Responses During EBV Infection

Numerous studies using antibiotic-treated and germ-free mice have declared that commensal microbiota could influence the generation of a diverse spectra of adaptive cells, such as virus-specific T cells and B cells. It was reported that the numbers of virus-specific CD4+ and CD8+ T cells toward hepatitis B virus (HBV), IAV, LCMV, and West Nile virus (WNV) were reduced in antibiotic-treated mice (Ichinohe et al., 2011; Abt et al., 2012; Chou et al., 2015; Jiang et al., 2017; Thackray et al., 2018). In the meantime, the virus-specific antibody response also diminished in the same series of studies in antibiotic-treated mice. As for the EBV infection, it remains to be explored whether the diminished virus-specific T/B cells would impair the control of EBV infection and result in EBV-driven cancers. In addition, there are fewer CD103^+^ DCs with impaired antigen-presenting capacity (a required site of antiviral CD8^+^ T cell priming) of naïve antibiotic-treated mice (Ichinohe et al., 2011; Thackray et al., 2018). Moreover, NK cells of antibiotic treated mice were impaired in the production of IFN-γ and cell-mediated cytotoxicity despite the maintenance, resulting in increased virus titers during murine cytomegalovirus infection (MCMV) (Ganal et al., 2012). The gut microbiota reconstitution for germ-free mice successfully protected mice from IAV and LCMV infection, suggesting an important role of microbiota in regulating pro-inflammatory cytokine responses during systemic virus infection (Ichinohe et al., 2011; Abt et al., 2012).

### Commensal Microbiota Could Promote the EBV Reactivation as well as EBV-Driven Cancers by Microbial-Derived Metabolites

Numerous studies have proposed that the diverse metabolites produced by host microbiome components might play a key role in the regulation of host health as well as cancer progression as reviewed by Cogdill et al. (2018). In the meantime, the concrete mechanisms behind the relationship between microbiome and cancer need further cautious explorations. The commensal microbia-derived metabolites play an important role in the regulation of host immunity (Levy et al., 2017) during the systemic virus infection. Recently, Steed et al. found that oral administration of the human-associated commensal gut bacteria *Clostridium orbiscendens* or its production, desaminotyrosine (DAT), could protect mice from lethal IAV infection through reduced immunopathology in the lung in a phagocyte-dependent process potentially through augmentation of the Type I IFN amplification loop  (Steed et al., 2017). The reactivation of EBV is known to be a vital step for the onset of EBV-related cancers. In addition to the coinfection of EBV and pathogens such as HIV, HPV, and others listed in Section 4, previous studies have reported an increased EBV replication in populations colonized with Gracilibacteria and Abiotrophia (Urbaniak et al., 2020). However, the key trigger for EBV reactivation and impairment of EBV control still remains to be explored.

It is reported that *P. gingivalis* and *F. nucleatum* can take part in the regulation of acetylation and deacetylase for histone, thus reactivating the EBV by modulating the BZLF1 promoter in EBV-infected cells (Imai et al., 2012a; Imai et al., 2012c). Butyrate (BA), which belongs to a short-chain fatty acid (SCFA) family (Louis et al., 2010; Imai et al., 2012b), was reported to be excreted by *P. endodontalis* and *F. nucleatum* in EBV carriers, and leads to the occurrences of periapical periodontitis (Makino et al., 2018; Himi et al., 2020). In addition, studies have also declared that the intraperitoneal injection of BA in EBV-driven cancers accelerates the expression of ZEBRA expression and reactivate the lytic EBV replication (Westphal et al., 1999; Westphal et al., 2000). A significantly higher level of BA has also been observed in the saliva of EBV-infected patients with BZLF1 transcription induction and lysine acetylation of histone H3 (Koike et al., 2020). In a word, BA is possibly a key trigger for EBV switching to reactivation and priming the EBV-driven cancers. However, Whether the reduction of BA-producing bacteria in host could be a possible treatment for EBV-driven cancer and how to achieve it remain to be explored.

## The Regulation of Commensal Microbiota Could Be a Target for Treatment of EBV-Driven Cancers

As for the treatment of EBV-driven cancers, studies have tried to develop some EBV-targeted therapies in addition to traditional chemotherapy and radiotherapy. However, limit progressions have been made by targeting the deregulated signal pathway in EBV infection or focusing on the drugs targeting EBV antigens, such as EBNA1 (Thompson et al., 2010; Messick et al., 2019). Up to now, no vaccine against EBV has been made successfully (Dugan et al., 2019; Cai et al., 2021). The application of nucleoside analogs which could act during the lytic phase of EBV, such as ganciclovir and zidovudine, in addition to IL-2 and CAR-T treatment, worked well in EBV-positive PCNSL (Aboulafia et al., 2006; Dugan et al., 2019). For latent EBV-driven cancer, the induction of the EBV lytic phase could be a rationale chosen, and BA was reported to take effect in refractory EBV-driven lymphoma (Ghosh et al., 2007; Perrine et al., 2007). Therefore, whether there would be another microbial product that could be efficient in the treatment of EBV-driven cancer needs further exploration. In recent years, increasing evidence suggested that the microbiome could regulate the host responses toward anticancer therapy, including chemotherapy, radiation, and targeted therapy (Cogdill et al., 2018). A recent study of advanced colorectal cancer reported that the diversity of blood microbiota influences the host response to chemotherapy and adoptive T cell immunotherapy (Yang et al., 2021). Consequently, whether the regulation of microbiome constitution would favor the therapeutic efficiency in EBV-driven cancer also needs to be explored.

## Concluding Remarks

Over the past few years, the roles of the commensal microbiome in modulating host immunity have been studied (Brosschot and Reynolds, 2018; Neil and Cadwell, 2018). The function of the commensal microbiome ranges from aiding in metabolism to competing with invasive pathogens (Abt et al., 2012; Belkaid and Hand, 2014). EBV, as a member of the commensal microbiome, has also been reported to regulate the host immune system and interact with other components of the microbiome. Some of these interactions are considered to induce the reactivation of EBV. However, a more thorough understanding of the molecular mechanisms by which specific constituents of the commensal microbiota would promote the reactivation of EBV, as well as driven EBV-associated cancers, is in great need.

## Author Contributions

YW wrote the paper. HX, JH, and HC supported and supervised the manuscript revision. All of the authors discussed and commented on the manuscript. All authors contributed to the article and approved the submitted version.

## Funding

This research was supported by funds from the National Natural Science Foundation of China (31701207, 82000175) and the Chen Xiao-ping Foundation for the Development of Science and Technology of Hubei Province.

## Conflict of Interest

The authors declare that the research was conducted in the absence of any commercial or financial relationships that could be construed as a potential conflict of interest.

## Publisher’s Note

All claims expressed in this article are solely those of the authors and do not necessarily represent those of their affiliated organizations, or those of the publisher, the editors and the reviewers. Any product that may be evaluated in this article, or claim that may be made by its manufacturer, is not guaranteed or endorsed by the publisher.
